# Supplementary material for: Real-World Outcomes for Localised Gastro-Oesophageal Adenocarcinoma Cancer Treated with Perioperative FLOT and Prophylactic GCSF Support in a Single Asian Centre
Source: Cancers (Basel). 2024 Nov 1;16(21):3697. doi: 10.3390/cancers16213697 (PMC11545039; doi:10.3390/cancers16213697)
Supplement: Supplementary file 1 [file cancers-16-03697-s001.zip › cancers-3246775-supplementary.pdf]

Supplementary Information

Table S1. Data on perioperative FLOT in a cohort (n = 56) of resectable GEA patients

	Patients<br>N (%)
**Number of cycles of pre-operative FLOT**	
Median (IQR)	4 (4 - 4)
Range	1 - 8
**Number of cycles of post-operative FLOT**	
Median (IQR)	4 (4 - 4)
Range	1 - 5
**Number of cycles of pre- and post-operative FLOT combined**	
Median (IQR)	8 (4 - 8)
Range	1 - 8
**Number of cycles of FLOT completed**	
1	3 (5.4)
2	2 (3.6)
4	9 (16.1)
5	2 (3.6)
6	3 (5.4)
7	4 (7.1)
8	33 (58.9)

Table S2. Odds ratio of patient and tumour characteristics for all AEs

	Event/No	Univariable OR (95% CI)	*p*-value
**Age**			
< 65	29/31	1	
≥ 65	22/25	0.51 (0.06-3.30)	0.476
**Ethnicity**			
Chinese	44/48	1	
Malay	3/3	NE	
Indian	4/5	0.36 (0.04-8.01)	0.412
**BMI (kg/m^2)**			
⩾23	20/22	1	
18.5-22.9	27/30	0.90 (0.11-5.92)	0.913
< 18.5	4/4	NE	
**Housing index**			
Low (1-2)	37/41	1	
Medium (3-4)	4/5	0.43 (0.05-9.55)	0.498
High (⩾5)	10/10	NE	
**ECOG**			
0	42/44	1	
1	9/12	0.14 (0.02-0.98)	0.048
**Significant weight loss (>10%)**			
Yes	16/17	1	

No	35/39	0.55 (0.03-4.07)	0.602
**Tumour site**			
Stomach	21/23	1	
GEJ	30/33	0.37 (0.05-3.05)	0.959
**Signet ring cells**			
Not specified	41/44	1	
Yes	10/12	0.37 (0.05-3.05)	0.304
**cN**			
N1-N3	42/43	1	
N0	9/13	0.05 (0.00-0.41)	0.013
**Clinical stage**			
III	39/41	1	
II	12/15	0.21 (0.02-1.37)	0.103
**Grading**			
3	24/27	1	
2	9/11	0.56 (0.08-4.80)	0.562
1	5/5	NE	
**Resection margin**			
R1	9/10	1	
R0	36/39	1.33 (0.06-11.91)	0.813
R2	1/1	NE	
**Major post-operative complications**			
G3 and above	12/13	1	
Nil	35/38	0.97 (0.05-8.45)	0.981
**ypN**			
N0	21/22	1	
N1-N3	27/30	0.43 (0.02-3.62)	0.477
**Pathological stage**			
0-II	29/30	1	
III-IV	19/22	0.22 (0.01-1.85)	0.202
**pCR**			
Yes	11/12	1	
No	40/44	0.91 (0.04-6.97)	0.935

Table S3. Odds ratio of patient and tumour characteristics for G3-4 AEs

	Event/No	Univariable OR (95%CI)	p-value
**Age**			
≥ 65	7/25	1	
< 65	3/31	0.28 (0.05-1.13)	0.087
**Sex**			
Female	3/9	1	
Male	7/47	0.35 (0.07-1.95)	0.199
**Ethnicity**			
Chinese	8/48	1	
Malay	1/3	2.50 (0.11-29.37)	0.476

Indian	1/5	1.25 (0.06-9.97)	0.850
**BMI**			
< 18.5	1/4	1	
18.5-22.9	7/30	0.91 (0.10-20.09)	0.941
⩾23	2/22	0.30 (0.02-7.61)	0.380
**Housing index**			
Low (1-2)	4/10	1	
Medium (3-4)	4/41	0.16 (0.03-0.84)	0.029
High (⩾5)	2/5	1.00 (0.10-9.12)	1.000
**Significant weight loss (>10%)**			
Yes	4/17	1	
No	6/39	0.59 (0.14-2.63)	0.467
**Tumour site**			
Stomach	7/23	1	
GEJ	3/33	0.23 (0.04-0.94)	0.051
**Signet ring cells**			
Not specified	9/44	1	
Yes	1/12	0.35 (0.02-2.21)	0.349
**cN**			
N0	3/13	1	
N1-N3	7/43	0.65 (0.15-3.42)	0.577
**Clinical stage**			
II	3/15	1	
III	7/41	0.82 (0.19-4.29)	0.800
**Grading**			
3	6/27	1	
2	2/11	0.78 (0.10-4.18)	0.782
1	1/5	0.87 (0.04-7.48)	0.912
**Major post-operative complications**			
3 and above	5/13	1	
Nil	4/38	0.19 (0.04-0.86)	0.032
**ASA score**			
2	5/28	1	
3	5/25	1.15 (0.28-4.70)	0.842
**ypN**			
N0	4/22	1	
N1-N3	6/30	1.13 (0.28-4.96)	0.869
**Pathological stage**			
0-II	6/30	1	
III-IV	4/22	0.89 (0.20-3.58)	0.869

Table S4. Surgical and pathological findings of operated GEA patients (n=52)

	Patient No. (%) (n=52)
**Type of surgery**	
Total gastrectomy	18 (34.6)
Subtotal gastrectomy	10 (19.2)
Oesophagectomy	20 (38.4)
Total gastrectomy with distal oesophagectomy	1 (1.9)
Others	3 (5.8)
**Pathological stage**	
0	8 (15.4)
I	6 (11.5)
II	16 (30.8)
III	21 (40.4)
IV	1 (1.9)
**ypT**	
T0	8 (15.4)
T1	5 (9.6)
T2	6 (11.5)
T3	24 (46.2)
T4	9 (17.3)
**ypN**	
N0	22 (42.3)
N1	12 (23.1)
N2	5 (9.6)
N3	13 (25.0)
**Resection margin**	
R0	39 (75.0)
R1	10 (19.2)
R2	1 (1.9)
Unknown	2 (3.8)
**AJCC/CAP tumour regression grading**	
0, complete response	8 (15.4)
1, near complete response	2 (3.8)
2, partial response	25 (48.1)
3, poor or no response	10 (19.2)
Unknown	7 (13.5)
**Number of LN dissected**	
Median (IQR)	22.0 (16.5 - 37.0)
Range	4.0 - 63.0
**Duration of hospitalization, days**	
Median (IQR)	9.5 (8.0 - 14.0)
Mean (SD)	13.4 (11.3)
Range	4.0 - 72.0
Unknown	2 (3.8)

Table S5. Comparison between median weight at different treatment phases (Wilcoxon signed rank test) in a cohort (n = 56) of resectable GEA patients

		Difference in median weight, kg (95% CI)	p-value
Diagnosis	Pre-operative	0.1 (-0.9 to 1.1)	0.839
	Post-operative	-1.2 (-2.9 to 0.5)	0.153
	Post-treatment	-4.2 (-6.3 to -2.0)	<0.001
Pre-operative	Post-operative	-1.4 (-2.7 to -0.1)	0.032
	Post-treatment	-5.5 (-7.1 to -3.8)	<0.001
Post-operative	Post-treatment	-3.7 (-5.1 to -2.4)	<0.001

Table S6. Univariate analysis for DFS

	All patients (N = 56)			Surgical population (N = 52)		
	Event/No	Univariable HR (95% CI)	p-value	Event/No	Univariable HR (95% CI)	p-value
**Age**						
< 65	11/31	1		11/30	1	
⩾ 65	9/25	1.00 (0.43-2.51)	0.940	7/22	0.81 (0.31-2.10)	0.663
**Sex**						
Male	19/47	1		17/43	1	
Female	1/9	0.23 (0.03-1.75)	0.157	1/9	0.26 (0.03-1.95)	0.190
**Ethnicity**						
Chinese	17/48	1		15/44	1	
Malay	2/3	1.81 (0.41-7.93)	0.430	2/3	2.06 (0.46-9.13)	0.342
Indian	1/5	0.61 (0.08-4.61)	0.633	1/5	0.69 (0.09-5.29)	0.724
**BMI at diagnosis, kg/m²**						
⩾ 23	7/22	1		6/21	1	
18.5-22.9	10/30	1.20 (0.45-3.16)	0.430	9/27	1.29 (0.46-3.63)	0.634
< 18.5	3/4	2.68 (0.69-10.44)	0.633	3/4	3.21 (0.79-12.95)	0.102
**Housing index**						
High (⩾ 5)	4/10	1		3/9	1	
Medium (3-4)	14/41	1.00 (0.32-3.05)	0.995	13/39	1.27 (0.36-4.50)	0.711
Low (<3)	2/5	1.49 (0.27-8.33)	0.652	2/4	2.10 (0.34-12.95)	0.425
**ECOG**						
0	13/44	1		11/41	1	
1	7/12	2.28 (0.91-5.75)	0.080	7/11	2.61 (1.00-6.77)	**0.039**
**Tumour site**						
GEJ	14/33	1		13/31	1	
Stomach	6/23	0.44 (0.17-1.15)	0.095	5/21	0.38 (0.13-1.08)	0.069
**MMR**						
pMMR	15/41	1		13/38	1	
dMMR	1/2	2.08 (0.27-16.21)	0.485	1/2	2.57 (0.32-20.42)	0.373
**Signet ring cells**						
Not specified	17/44	1		15/41	1	

Yes	3/12	0.64 (0.19-2.17)	0.471	3/11	0.72 (0.21-2.49)	0.604
**Lauren's type**						
Intestinal	5/13	1		4/12	1	
Diffuse	1/2	1.02 (0.12-8.70)	0.993	1/2	1.24 (0.14-11.18)	0.848
Mixed	2/6	0.58 (0.11-3.04)	0.522	2/6	0.71 (0.13-3.91)	0.691
Not evaluable	12/35	0.81 (0.28-2.32)	0.698	11/32	0.92 (0.29-2.93)	0.894
**cN**						
N0	3/13	1		3/12	1	
N1-N3	17/43	1.66 (0.49-5.67)	0.418	15/40	1.47 (0.42-5.07)	0.544
**Clinical stage**						
II	3/15	1		3/15	1	
III	17/41	2.67 (0.78-9.12)	0.118	15/37	2.41 (0.69-8.35)	0.166
**Grading**						
3	9/27	1		9/26	1	
2	7/11	1.82 (0.68-4.89)	0.236	7/11	1.81 (0.67-4.87)	0.240
1	1/5	0.40 (0.05-3.17)	0.385	1/5	0.38 (0.05-3.05)	0.366
**4 cycles full-dose FLOT**						
No	8/30	1		8/28	1	
Yes	12/26	1.96 (0.80-4.81)	0.119	10/24	1.67 (0.66-4.24)	0.279
**8 cycles full-dose FLOT**						
No	12/35	1		10/31	1	
Yes	8/21	0.97 (0.40-2.38)	0.953	8/21	1.16 (0.46-2.95)	0.753
**Significant weight loss (>10%)**						
No	16/39	1		14/35	1	
Yes	4/17	0.42 (0.14-1.25)	0.117	4/17	0.46 (0.15-1.41)	0.176
**Resection margin**						
R1				5/10	1	
R0				13/39	0.34 (0.12-0.98)	**0.045**
R2				0/1	NE	
**ypT**						
T1-T4				17/44	1	
T0				1/8	0.28 (0.04-2.13)	0.220
**ypN**						
N1-N3				16/30	1	
N0				2/22	0.10 (0.02-0.45)	**0.003**
**Pathological stage**						
0-II				8/30	1	
III-IV				10/22	3.04 (1.15-8.09)	**0.026**
**pCR**						
No				17/44	1	
Yes				1/8	0.28 (0.04-2.13)	0.220
**Major post-operative complications**						
⩾ G3				6/13	1	
<G3 or none				11/38	0.52 (0.19-1.40)	0.196
**ASA score**						

2				9/27	1	
3				9/25	1.66 (0.65-4.28)	0.293
**AJCC/CAP tumour regression grading**						
3				4/10	1	
2				11/25	0.60 (0.19-1.93)	0.390
1				0/2	NE	
0				1/8	0.16 (0.02-1.43)	0.100

Figure S1. Kaplan-meier curves for DFS and OS

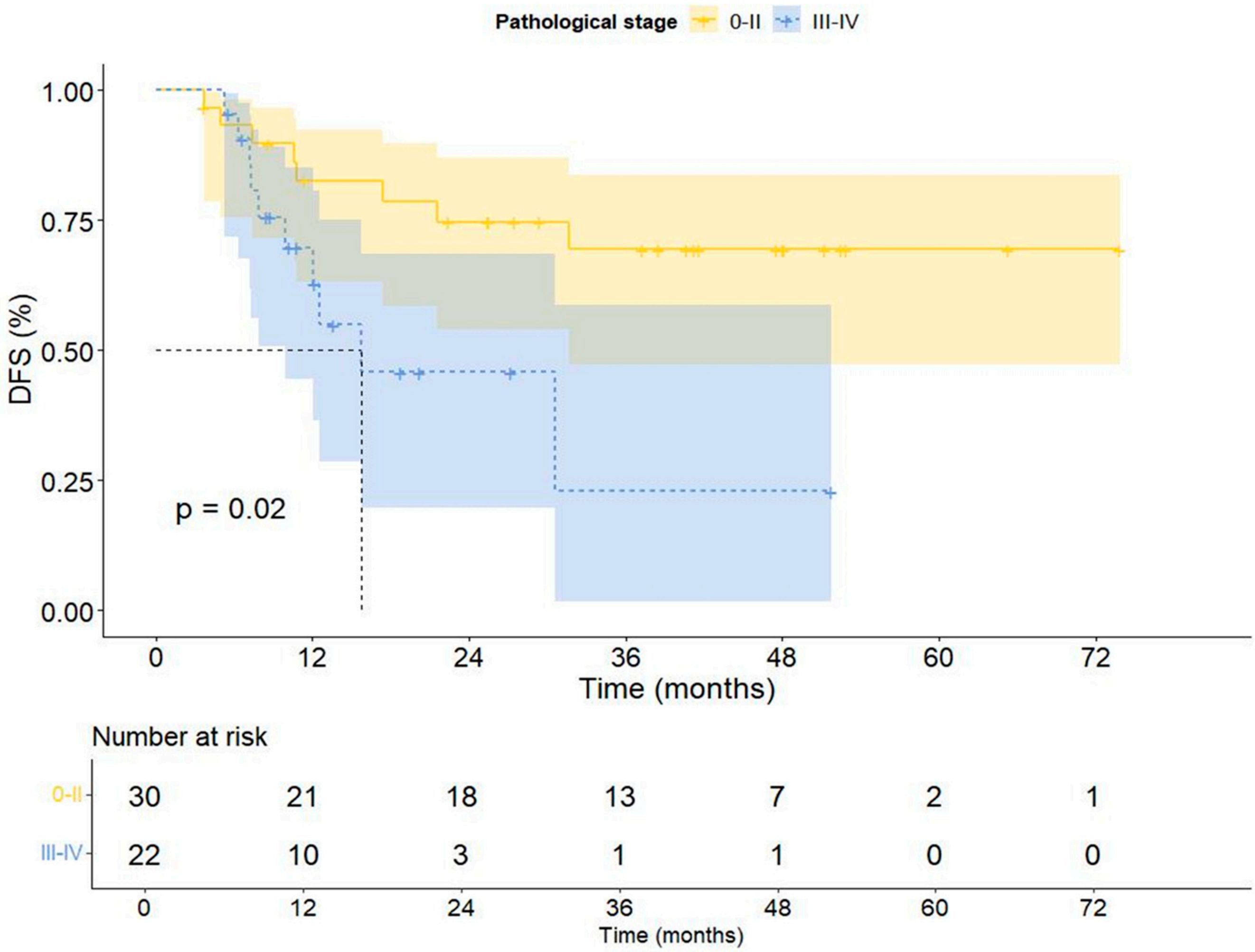

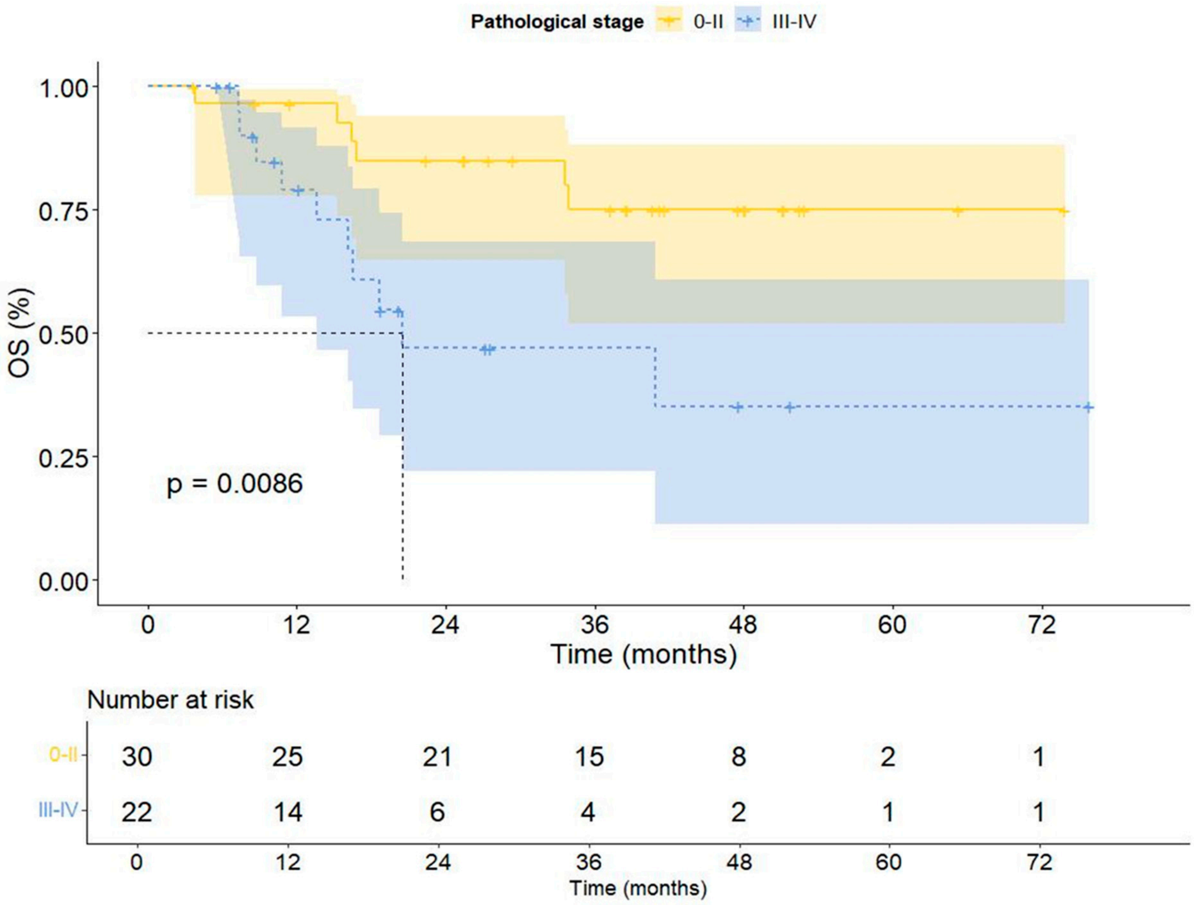

Pathological stage
0-II
III-IV
1.00
0.75
0.50
0.25
0.00
OS (%)
p = 0.0086
0
12
24
36
48
60
72
Time (months)
Number at risk
0-II
III-IV
30 25 21 15 8 2 1
22 14 6 4 2 1 1
0
12
24
36
48
60
72
Time (months)

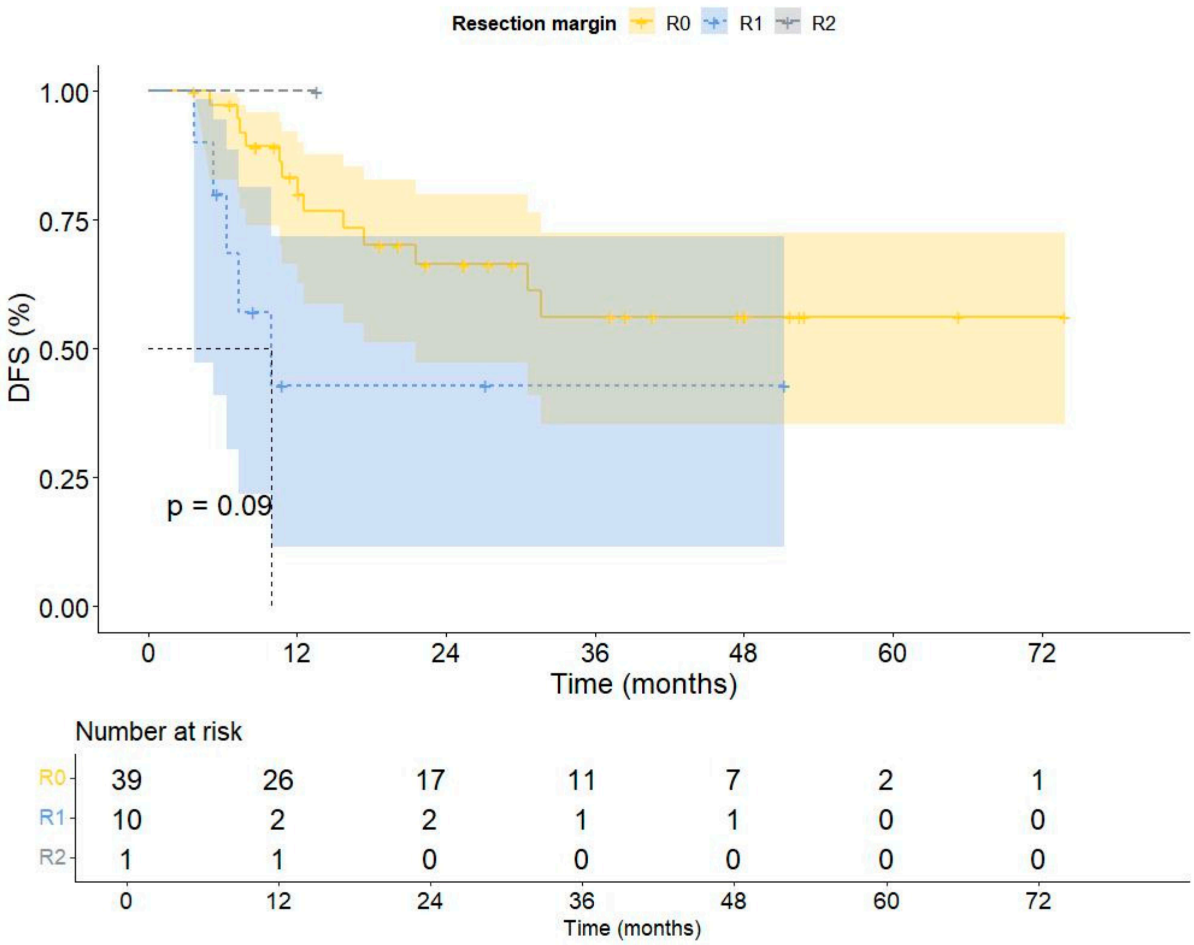

Resection margin
R0
R1
R2
1.00
0.75
0.50
0.25
0.00
DFS (%)
p = 0.09
0
12
24
36
48
60
72
Time (months)
Number at risk
R0 39 26 17 11 7 2 1
R1 10 2 2 1 1 0 0
R2 1 1 0 0 0 0 0
0
12
24
36
48
60
72
Time (months)

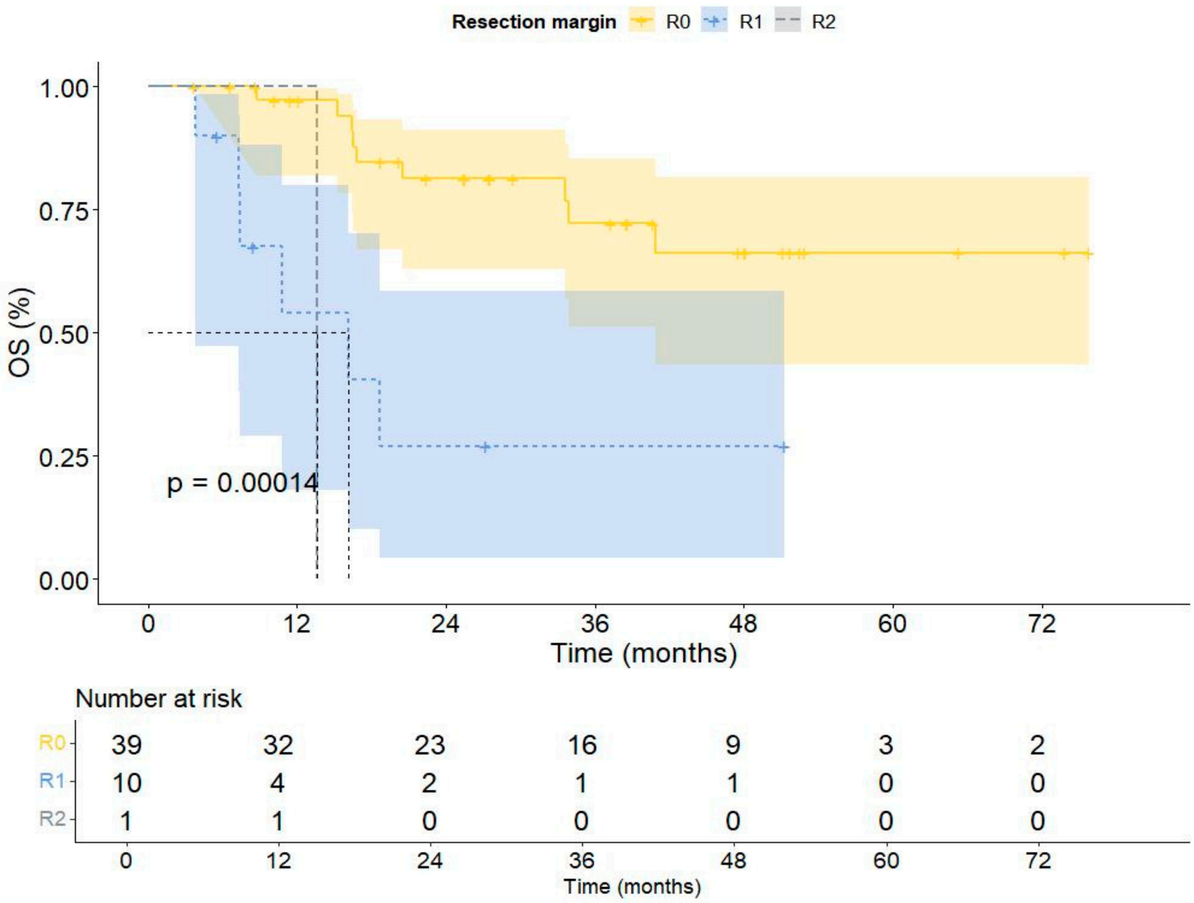
Resection margin
R0
R1
R2
1.00
0.75
0.50
0.25
0.00
OS (%)
p = 0.00014
0
12
24
36
48
60
72
Time (months)
Number at risk
R0 39 32 23 16 9 3 2
R1 10 4 2 1 1 0 0
R2 1 1 0 0 0 0 0
0
12
24
36
48
60
72
Time (months)
